# By-design molecular architectures *via* alkyne metathesis

**DOI:** 10.1039/d1sc01881g

**Published:** 2021-05-22

**Authors:** Shaofeng Huang, Zepeng Lei, Yinghua Jin, Wei Zhang

**Affiliations:** Department of Chemistry, University of Colorado Boulder 80309 USA wei.zhang@colorado.edu

## Abstract

Shape-persistent purely organic molecular architectures have attracted tremendous research interest in the past few decades. Dynamic Covalent Chemistry (DCvC), which deals with reversible covalent bond formation reactions, has emerged as an efficient synthetic approach for constructing these well-defined molecular architectures. Among various dynamic linkages, the formation of ethynylene linkages through dynamic alkyne metathesis is of particular interest due to their high chemical stability, linearity, and rigidity. In this review, we focus on the synthetic strategies of discrete molecular architectures (*e.g.*, macrocycles, molecular cages) containing ethynylene linkages using alkyne metathesis as the key step, and their applications. We will introduce the history and challenges in the synthesis of those architectures *via* alkyne metathesis, the development of alkyne metathesis catalysts, the reported novel macrocycle structures, molecular cage structures, and their applications. In the end, we offer an outlook of this field and remaining challenges.

## Abstract 2

The recent synthesis of novel shape-persistent 2D and 3D molecular architectures *via* alkyne metathesis is reviewed and the critical role of catalysts is also highlighted.

## Graphical abstract

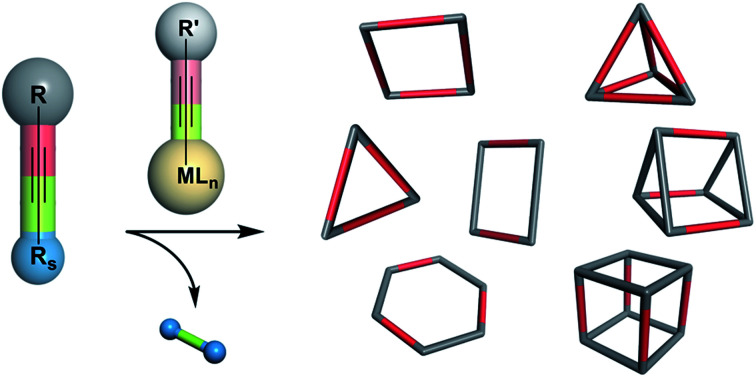

## 1. Introduction

In recent years, shape-persistent molecular architectures have attracted huge interest in supramolecular and materials chemistry. Due to their unique geometries and shape-persistency, these well-defined molecular architectures exhibit great potential in serving as rigid hosts for guest molecule recognition and separation. In the design of these architectures, the chemistry of the linkages should be judiciously chosen to obtain desired structures in high yields. Dynamic Covalent Chemistry (DCvC),^1,2^ which involves reversible covalent bond formation (activated under certain external stimuli, such as heat, catalyst, *etc.*), has been widely used in the construction of ordered structures. Compared with irreversible reactions (*e.g.*, cross-coupling, S_N_2 reaction, *etc.*), the reversible feature of DCvC enables bond exchange and self-correction during the reaction process and can provide thermodynamically stable products in high yields at equilibrium. On the other hand, to enhance the stability of molecular architectures, the robustness of the linkages should be considered. Carbon–carbon triple bonds are stable yet reversible and do not have *Z*/*E* isomer issues, which result in multiple isomers that are hard to isolate and characterize. Due to their rigid and linear geometry feature, alkyne bonds have been frequently employed in the synthesis of shape-persistent architectures over the past decades. Alkyne metathesis^3^ has been known as an important bond exchange reaction that can redistribute alkyne groups through reversible cleavage and reformation of carbon–carbon triple bonds. A broad range of shape-persistent architectures have been synthesized through alkyne metathesis under thermodynamically controlled conditions thanks to its reversibility and self-correction behavior. In this review, we discuss shape-persistent molecular architectures that have been synthesized using alkyne metathesis as the key step. Although total synthesis of natural products and polymer synthesis through alkyne metathesis are equally important, we will not cover those topics here. The history of the synthetic strategy, the development of alkyne metathesis catalysts, and the thermodynamic/kinetic control of the product formation are discussed in Section 2. The synthesis of shape-persistent macrocycles and cages is covered in Sections 3 and 4, respectively. The applications of those architectures are briefly summarized in Section 5. Finally, we provide conclusions of current research and outlook of future research directions in Section 6.

## 2. Overview of the synthetic strategy

In this section, the current progress in the development of alkyne metathesis catalysts, the strategies of shifting the equilibrium toward product formation, and the kinetic and thermodynamic aspects of the reaction pathways are discussed. The selection of catalysts is critical for the success of the complex molecular architecture synthesis. Catalysts with high activity and long lifetime are highly desired to allow the free exchange of alkyne partners among reaction components and reach the equilibrium with overall energy minimum. Functional group tolerance and user-friendliness are also important parameters that need to be considered. Since alkyne metathesis is an equilibrium reaction, removal of byproducts is necessary to shift the equilibrium toward the product formation. Removal of small alkynes through evaporation or adsorption, or removal of insoluble byproducts through precipitation have been common strategies to achieve a high conversion. Although alkyne metathesis is a reversible reaction and generally provides thermodynamically stable products at equilibrium, kinetic aspects of the reaction process, which often lead to unexpected yet intriguing structures, should not be overlooked. The introduction of kinetic factors into the thermodynamically controlled system could enable the formation of molecular architectures that are otherwise hard to obtain.

### 2.1. Alkyne metathesis catalysts

Ever since the discovery of alkyne metathesis in 1968,^3^ it has been widely applied in the synthesis of natural products^4–8^ and polymers.^9–14^ However, the synthesis of discrete shape-persistent ethynylene-linked molecular architectures, which particularly requires the high efficiency of a catalyst as well as suitable driving-force toward desired structures, has been a challenging task. According to the principle of DCvC, a highly active catalyst is crucial for various intermediates and/or side products to overcome the energy barriers, equilibrate into one another, and eventually form the desired product.

**Fig. 1:**
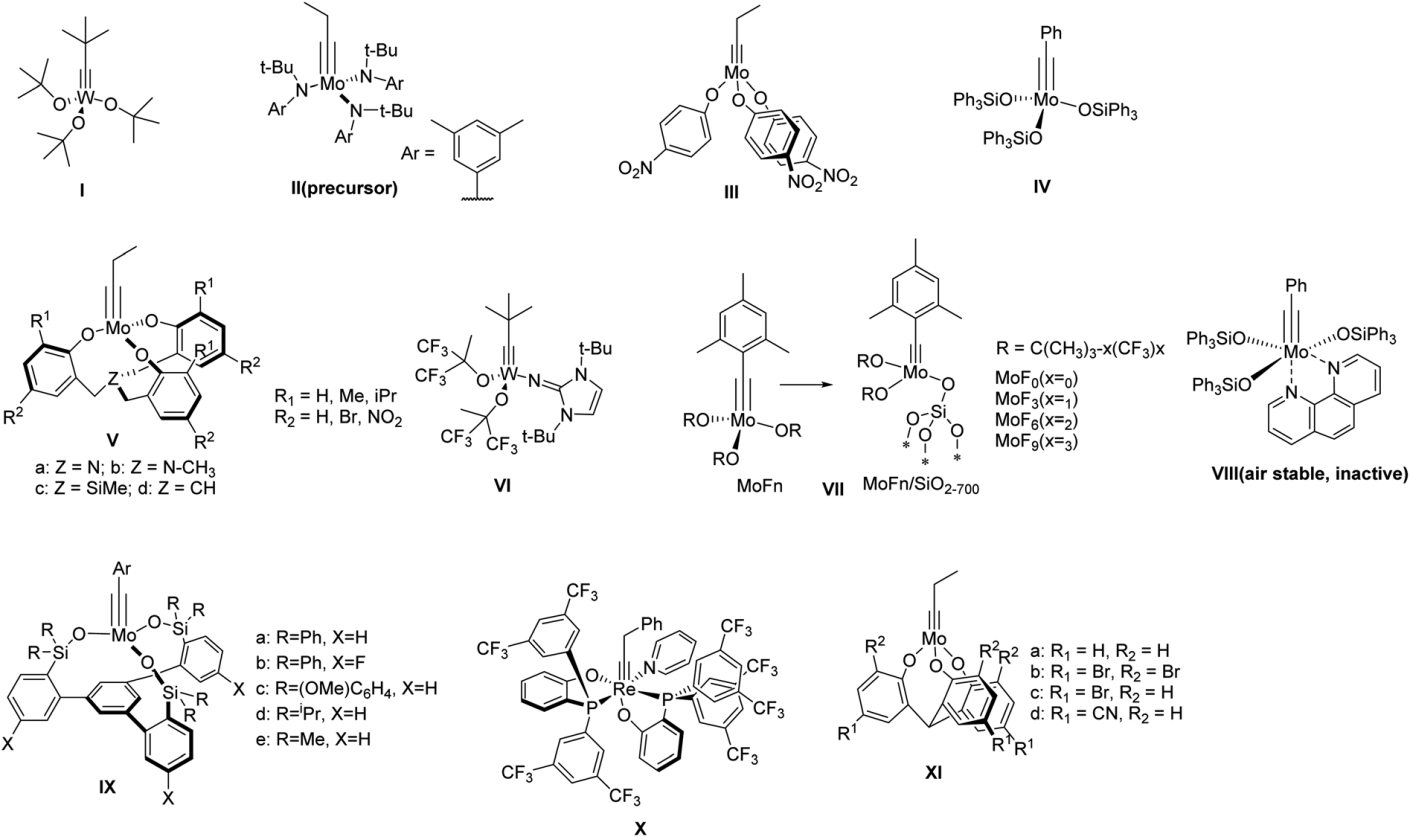
Representative structures of alkyne metathesis precursor and catalysts.

Within the past few decades, a variety of group VI metal-based complexes have been developed for alkyne metathesis. However, only a few of them have been utilized in the one-step synthesis of ethynylene-linked molecular architectures, mainly due to their relatively low activity and limited functional group compatibility. In 2000, Bunz and co-workers synthesized a cyclohexameric *m*-phenyleneethynylene macrocycle for the first time through alkyne metathesis using Mo(CO)_6_/phenol catalyst system albeit in only 0.5–6% yields.^15^ In 2003, Vollhardt and co-workers synthesized a series of trimeric phenyleneethynylene macrocycles with Schrock's catalyst (Fig. 1, structure **I**).^16^ However, except for the unsubstituted substrate, only 12–28% yields were obtained for other tested substrates, with some of them even non-reactive. It was not until 2004 when Moore and co-workers developed a highly active catalyst (Fig. 1, structure **III**) generated *in situ* from molybdenum-trisamide alkylidyne precursor (Fig. 1, structure **II**)^17,18^ that ethynylene-linked macrocycles can be synthesized through alkyne metathesis in one step in a high yield. The triarylsilanolate-based catalyst developed by Fürstner and co-workers (Fig. 1, structure **IV**) also showed good catalytic activity in synthesizing tetrameric carbazole-based macrocycle.^19^ The catalyst (**IV**) containing Ph_3_SiOH,^19,20^ a commercially available siloxy-based monodentate ligand, has also been applied in the synthesis of molecular cages in high yields upon *in situ* mixing the ligand with Mo precursor (**II**). However, the substrates with electron-withdrawing or chelating groups still need more active catalysts, *e.g.*, multidentate ligand-based catalysts.^21,22^ Zhang and co-workers developed a series of multidentate ligands consisting of trisbenzylamine, trisbenzylsilane, or trisbenzylmethine (Fig. 1, structure **V**),^23–26^ which exhibit high activity as well as broad substrate scope. With these multidentate ligand-based catalysts, many aryleneethynylene molecular cages have been successfully synthesized, which was not possible with the catalysts consisting of monodentate ligands (*e.g.*, **III**).^21,22,27^

There have been considerable efforts to develop user-friendly catalysts that are less sensitive to moisture and air. Tamm and co-workers reported one of the first catalysts, tungsten-based catalysts with imidazoline-2-iminato ligands,^28,29^ which can operate efficiently at room temperature with low catalyst loading (Fig. 1, structure **VI**). Later, they also reported a series of fluorinated alkoxide-based molecular (MoF_*n*_) and silica-supported (MoF_*n*_/SiO_2–700_) catalysts (Fig. 1, structure **VII**),^30,31^ which can catalyze the metathesis of terminal alkynes under mild conditions. Fürstner and co-workers developed a series of triarylsilanolate-based catalysts (Fig. 1, structure **IV** & **VIII**).^19,20^ Among them, the phenanthroline-stabilized complex (**VIII**), although inactive, can be stored under open-air condition and becomes active upon heating in the presence of MnCl_2_. In 2019, Fürstner and Lee independently reported a multidentate silanolate catalyst (Fig. 1, structure **IX-a**),^32,33^ which exhibits moderate tolerance towards air and water, as well as better functional group compatibility. Later, Fürstner and co-workers developed a series of such “canopy” type catalysts (Fig. 1, structure **IX**),^34^ which exhibit high catalytic activity in technical-grade solvents without rigorous drying and purification prior to use. In 2020, Jia and co-workers reported a d^2^Re(v)-based catalyst bearing the PO-chelating ligands and a pyridine ligand that can be stored under open-air conditions (Fig. 1, structure **X**).^35^ The catalyst was active for various substrates including carboxylic acids at high temperature under nitrogen. Very recently, Zhang and co-workers developed multidentate tris(2-hydroxyphenyl)methine-based catalysts (Fig. 1, structure **XI**),^36^ which can catalyze the reaction under open-air conditions for a broad range of substrates. The active species dispersed in paraffin wax can be stored on benchtop for 30 days with only small decrease in activity. Although most of these catalysts are yet to be tested toward the synthesis of discrete molecular architectures, they show great potential in developing user-friendly catalysts for more widespread applications.

### 2.2. Byproduct removal

**Fig. 2:**
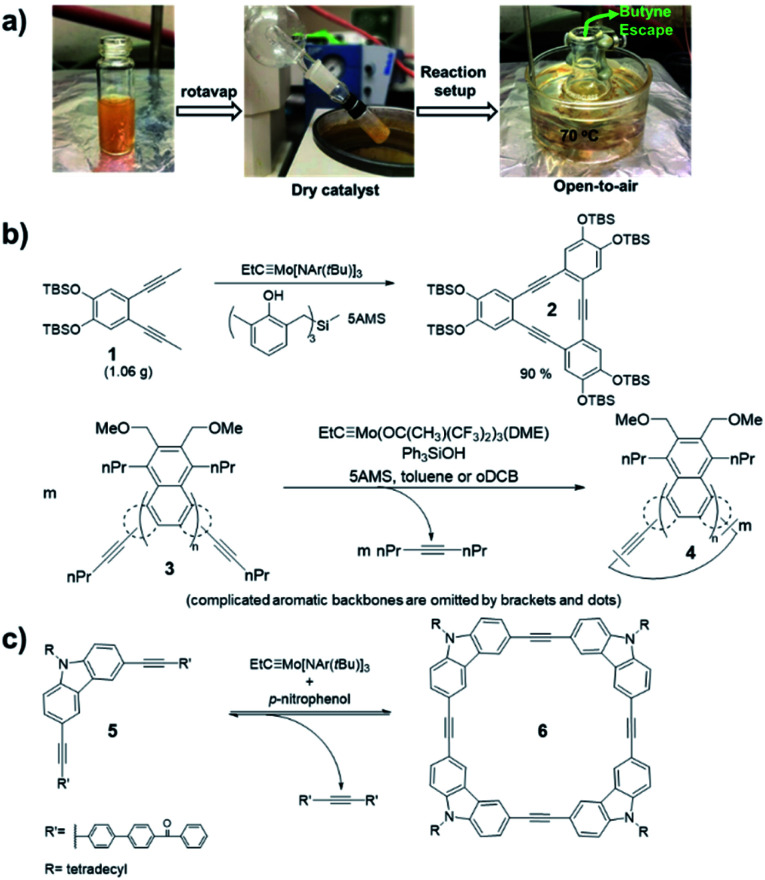
Representative driving-force in alkyne metathesis: (a) evaporation-driven; (b) adsorption-driven; (c) precipitate-driven. Reproduced with permission: (a) from ref. 36, Copyright 2021, Springer Nature; (b) from ref. 38, Copyright 2013, Wiley; from ref. 39, Copyright 2020, The Royal Society of Chemistry; (c) from ref. 41, Copyright 2004, The American Chemical Society.

Alkyne metathesis is an equilibrium reaction. In order to push the equilibrium toward product formation, byproducts have to be removed. There have been two main ways to remove byproducts: (1) removal of small alkynes by evaporation or adsorption; (2) removal of the large insoluble alkynes through precipitation. Initially, the vacuum-driven strategy was developed for removal of 2-butyne byproduct in macrocycle synthesis, which could poison the catalyst.^15,37^ However, it was found that the method was only limited to milligram-scale synthesis due to the low efficiency in the byproduct removal and the easy introduction of air/moisture to the reaction system, causing the catalyst deactivation. Recently, Zhang and co-workers demonstrated open-air alkyne metathesis using a highly active catalyst (**XI-d**), where the 2-butyne byproduct can quickly evaporate out of the reaction system and the carbazole cyclic tetramer **6** was obtained in 98% yield (Fig. 2a).^36^ In 2010, Fürstner and co-workers first used 5Å molecular sieves (5Å MS) to trap the byproduct 2-butyne.^19^ This strategy could achieve the gram-scale synthesis of aryleneethynylene macrocycles **2** (Fig. 2b).^38^ However, the installation of propynyl group is not very convenient: the propyne gas is hard to handle quantitatively, which may cause explosion upon heating in a closed system; the saturated solution of propyne in common organic solvents is too dilute (∼5%), causing the dilution of the whole reaction; the organometallic reagents prepared from propyne (*e.g.*, propynyl lithium, Grignard reagent), although easier to handle and could be used in a concentrated solution, are not compatible with electrophiles or protic substrates. These drawbacks can be overcome by using 1-pentynyl group as terminal groups (*e.g.*, **3**, Fig. 2b). In 2019, Tilley and co-workers reported such an example in the synthesis of macrocycle **4**, where the byproduct, 4-octyne, could be trapped by 5Å MS.^39,40^ Compared with propyne, 1-pentyne is a liquid that is much easier to handle quantitatively, also with a higher solubility. The 1-pentynyl group could be installed *via* Sonogashira coupling reaction, which is robust as well as selective toward different halides.

Removal of byproducts through precipitation is another widely used approach. Moore and co-workers first developed the precipitate-driven strategy,^41^ in which the benzoylbiphenyl (PPT)-substituted monomers **5** were used (Fig. 2c). By utilizing this strategy, the aryleneethynylene macrocycle **6** was successfully synthesized in gram-scale for the first time.^42^ Since the PPT terminal alkyne is easy to handle compared with propyne gas, it has been successfully used in synthesis of multiple molecular cages. However, such precipitate-driven strategy has low reaction atom-economy, wasting a lot of mass as precipitates, and monomers substituted with the precipitating groups often have a low solubility and high polarity.

### 2.3. Thermodynamically *vs.* kinetically controlled conditions

One of the important advantages of DCvC is the capability of yielding the most thermodynamically favored product predominantly at equilibrium. The bond angles and geometry of monomers need to be specifically designed so that the targeted structures have the energy preference. Such principle has been well-practiced in supramolecular chemistry as well as in DCvC. A plethora of complex 2D and 3D molecular architectures have been successfully synthesized through one-step dynamic assembly process under thermodynamically controlled conditions. As illustrated by Moore and co-workers in a systematic study on aryleneethynylene macrocycle formation,^43^ the dynamic assembly process toward the energy favored aryleneethynylene macrocyclic product generally follows two stages: a fast monomer-to-oligomer conversion followed by slow equilibration leading to the desired macrocyclic structures. Successful thermodynamically controlled reactions require free exchange of all the reactive components along the reaction pathway until reaching the equilibrium and energy minima of the dynamic system. However, oftentimes, there are kinetic traps, such as precipitation of reaction intermediates or formation of non-exchangeable intermediates, which eventually lead to kinetically preferred products. Although kinetic aspects in the thermodynamically controlled systems have largely been neglected, they could have intriguing contributions to the formation of “unusual” molecular architectures.

**Fig. 3:**
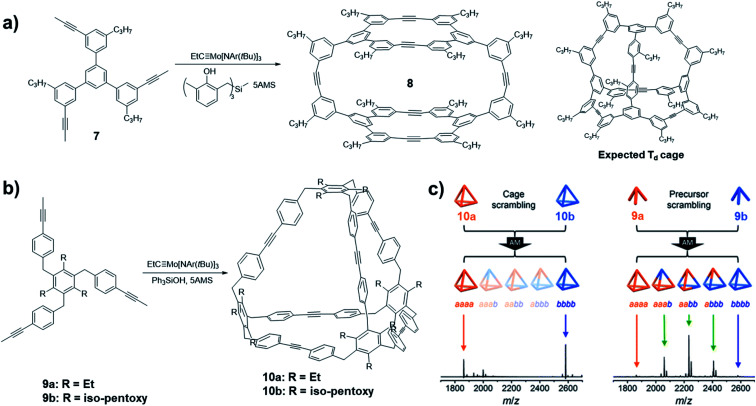
Examples of architecture synthesis bearing kinetic traps: (a) synthesis of *D*_2h_ symmetric cage **8** by Zhang and co-workers; (b) synthesis of *T*_d_ symmetric cage **10** by Moore and co-workers; (c) scrambling experiments conducted with cages **10** (left) and the monomers **9** (right). Reproduced with permission: (a) from ref. 44, Copyright 2014, Wiley; (b) and (c) from ref. 45, Copyright 2016, The American Chemical Society.

In 2014, Zhang and co-workers reported an example of the cage synthesis interfered by the kinetic factor.^44^ The monomer **7** was designed with the bond angles and direction of reactive terminal alkynes that match well with the geometry of a highly symmetrical *T*_d_ cage. However, unexpectedly, a cage structure with *D*_2h_ symmetry was obtained when the monomer was subjected to the alkyne metathesis in the presence of the catalyst (**V-c**, R_1_ = Me, R_2_ = H). The cage structure **8** was unambiguously solved by single crystal X-ray diffraction analysis (Fig. 3a). Based on the computational calculations, the experimentally isolated cage **8** has no energy preference compared to the originally targeted tetrahedron-shaped *T*_d_ symmetric cage. The mechanism study on the formation of the unexpected cage revealed that the readily formation of the macrocycle panel is the key step in the formation of the *D*_2h_ cage **8**. The top panel macrocycle can be isolated in 22% yield under alkyne metathesis condition even in a closed system without the addition of 5Å MS. Although the scrambling experiment showed that the cage **8** is not kinetically trapped, the favored reaction rate of macrocyclization largely influences the resulting product structure by hindering the conversion of the macrocyclic intermediate to other intermediates and the starting materials.

Oftentimes, the presence of a “kinetic trap” can provide closed molecular architectures in abnormally high yields without preorganization of monomers. In 2016, Moore and co-workers reported a kinetically trapped cage with *T*_d_ symmetry through one-step alkyne metathesis in an exceptionally high yield.^45^ They designed a tripodal monomer **9** but with more flexible arms and thus “loose” preorganization for the cage synthesis. The alkyne metathesis was performed in 1,2,4-trichlorobenzene, using Mo precursor (**II**) and commercially available Ph_3_SiOH as the ligand in the presence of 5Å MS. Both monomers with different alkyl substituents gave the tetrameric cages in excellent yields higher than 90% (Fig. 3b). The structure of cage **10** was confirmed by the single crystal analysis using the synchrotron X-ray diffraction. The authors demonstrated that one of the important reasons for the unanticipated nearly quantitative yield in the cage synthesis is the kinetic trapping. In the cage scrambling experiment, no mixed cages were observed on MALDI-TOF, supporting that the tetrahedral cages are kinetically trapped (Fig. 3c, left). By contrast, the precursor scrambling experiment showed mixed cage signals (Fig. 3c, right), which rules out the possibility of narcissistic self-sorting. Such “kinetic trap” can be attributed to the high effective molarity in the preorganized intermediate, which can rapidly undergo intramolecular ring closing rather than intermolecular reaction with another intermediate. Similar kinetic trapping has also been observed in imine-linked higher rung ladder molecules and the organic trefoil knot.^46,47^

## 3. Synthesis of shape-persistent aryleneethynylene macrocycles

Shape-persistent aryleneethynylene macrocycles are of great interest in the fields of supramolecular chemistry and materials chemistry due to their novel structures and potential applications in host–guest chemistry,^48–50^ liquid crystalline solids,^51^ and molecular channels.^52–54^ Before the utilization of alkyne metathesis, these macrocycles were synthesized through transition metal-catalyzed irreversible coupling reactions, which require multistep synthesis from monomers to oligomers and final cyclization with tedious purification and a low overall yield. In addition, due to the kinetically controlled nature of cross-coupling reactions, the reaction concentration of the final cyclization step has to be low to prevent intermolecular reaction and promote intramolecular cyclization, which is not efficient and generates a significant amount of solvent waste. Since the first report of gram-scale macrocycle synthesis *via* alkyne metathesis in 2004 by Moore and co-workers,^41^ the synthesis of shape-persistent aryleneethynylene macrocycles on large-scale under a regular concentration has come into reality. The synthesis of planar macrocycles has been well-developed and covered in various review articles.^6,55^ In this section, we will mainly discuss the synthesis of shape-persistent macrocycles by varying the geometry of the monomers, using the sterically hindered monomers and applying the post-synthetic approach. We will focus on introduction of novel functional groups into the macrocyclic architectures to bring other intriguing properties.

**Fig. 4:**
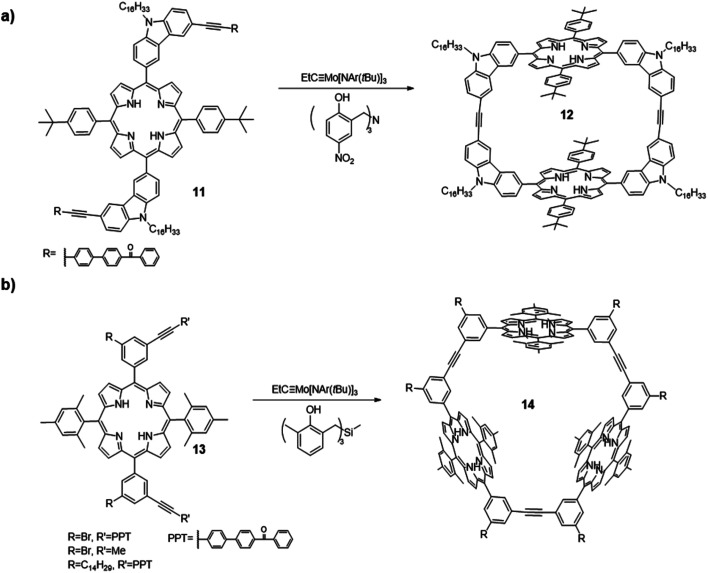
(a) Dimeric porphyrin-based macrocycle **12**; (b) trimeric porphyrin-based macrocycle **14** synthesized *via* one-step alkyne metathesis macrocyclization. Reproduced with permission: (a) from ref. 56, Copyright 2012, The Royal Society of Chemistry; (b) from ref. 57, Copyright 2016, The American Chemical Society.

In 2012, Zhang and co-workers synthesized a macrocycle containing two porphyrin units through alkyne metathesis (Fig. 4a).^56^ The macrocycle **12** was synthesized through dimerization of a porphyrin-based diyne monomer **11** using the catalyst (**V-a**, R_1_ = H, R_2_ = NO_2_). The PPT group was installed for the precipitation-driven alkyne metathesis. The dimerized macrocycle was obtained in a 60% isolated yield. Based on a similar design, cyclic porphyrin trimer **14** was synthesized from another porphyrin-based diyne monomers **13** with an angle of 60° between two end groups (PPT or propynyl groups, Fig. 4b).^57^ The catalyst (**V-c**, R_1_ = Me, R_2_ = H) was used and the macrocyclic products were isolated in high yields (80–83%). Precipitation-driven (from the monomer **13**, R = Br/C_14_H_29_, R′ = PPT) and adsorption-driven (from the monomer **13**, R = Br, R′ = Me) alkyne metathesis provided similar yields, indicating similar byproduct removal efficiencies can be achieved through these methods. The introduction of porphyrin moieties renders these macrocycles having good binding affinities toward fullerenes due to the well-defined internal cavities and favorable interactions between fullerene and porphyrins. In addition, when the porphyrin was metallated by zinc, the macrocycle **14** showed a strong binding affinity for a tripyridine guest molecule.

**Fig. 5:**
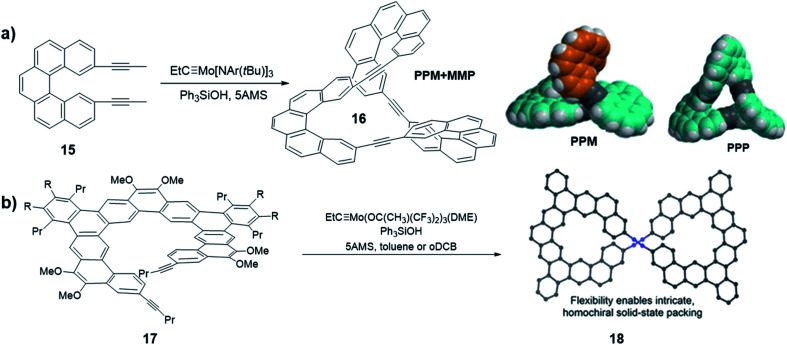
(a) Möbius ring structures synthesized by Moore and co-workers, the space-filling models of DFT-calculated structures of PPM and PPP are shown. (b) Möbius ring structures synthesized by Tilley and co-workers. Reproduced with permission: (a) from ref. 58, Copyright 2020, The American Chemical Society; (b) from ref. 40, Copyright 2020, The American Chemical Society.

As discussed previously, in thermodynamically controlled alkyne metathesis, monomer geometry and bond angles largely determine the product structures, which are energy favored with minimal angle strains. On the other hand, sterically hindered monomers can bring interesting kinetic aspects by raising the activation energy of intermediate species. In 2020, Moore and co-workers first used a sterically hindered helicene monomer **15** to efficiently synthesize a twisted Möbius ring (a topology with only one boundary curve and one side) structure **16** through one-step alkyne metathesis (Fig. 5a).^58^ The single crystal X-ray diffraction confirmed the twisted structure of the enantiomeric pair of PPM/MMP (P stands for a right-handed helix and M stands for a left-handed helix). Although energy calculations show that the PPP/MMM enantiomeric pair is the thermodynamically more stable product, the PPM/MMP enantiomeric pair was formed as the predominant product experimentally. By utilizing DFT calculation, the authors found there is 15.4 kcal mol^−1^ difference between the activation energy in the rate-determining step of PPM/MMP *vs.* PPP/MMM formation. The formation of the energy favored unstrained products has to overcome high energy barriers of the four-membered metallacycle intermediates, thus is kinetically prohibited. This example highlights the significance of kinetic aspects, which can open many exciting opportunities in forming intricate molecular architectures through DCvC.

Another interesting helicene was reported by Tilley and co-workers. A sterically hindered helicene monomer **17** was converted to a dimeric macrocycle *via* alkyne metathesis (Fig. 5b).^40^ The expanded helicene macrocycle **18** crystalizes in supramolecular helices with the uncommon, non-centrosymmetric space group *P*6_4_22, where all the molecules are homochiral. When crystallizing out from the solution phase, the two helical structures with opposite handedness (PP/MM) could form a double helix superstructure, in which the shortest π-stacking distance (3.5 Å) enhances the stability of the superstructure. The formation of chiral twisted structures from sterically hindered monomers currently remains largely unexplored. Chiral or pre-chiral monomers subjected to alkyne metathesis will afford novel structures with different combinations of chirality.

**Fig. 6:**
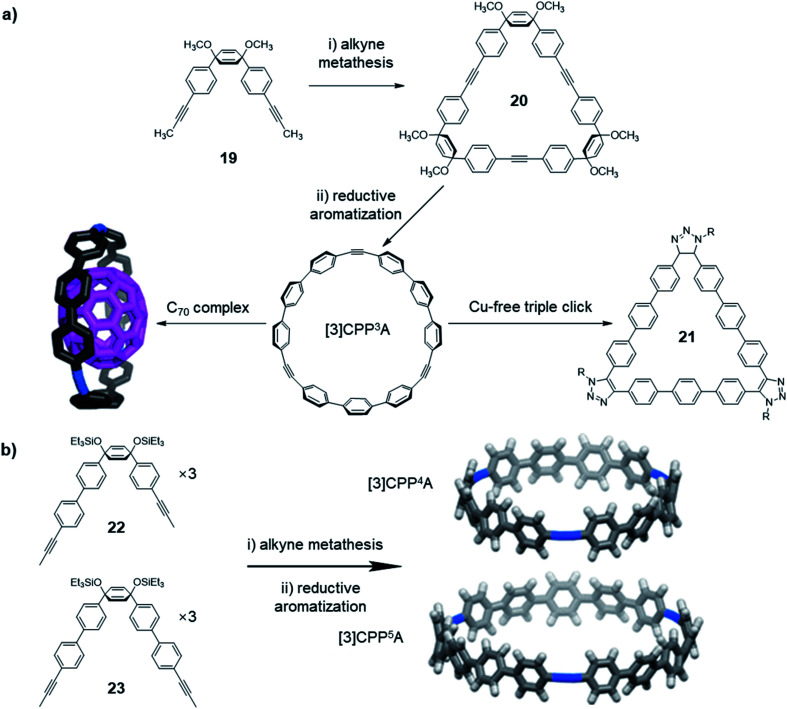
(a) Synthesis of [3]CPP^3^A, and its binding with C_70_ and occurring triple-click reaction. (b) Synthesis of [3]CPP^4^A and [3]CPP^5^A structures. Reproduced with permission: (a) from ref. 59, Copyright 2016, The American Chemical Society; (b) from ref. 60, Copyright 2019, The American Chemical Society.

In addition to the structures directly obtained through alkyne metathesis, the post-synthetic strategy has also been used in the synthesis of novel ethynylene-linked molecular belts, such as cycloparaphenyleneacetylene (CPPA). In 2016, Moore and co-workers first demonstrated the synthesis of CPPA *via* alkyne metathesis of cyclohexadienyl-containing monomer **19** followed by reductive aromatization (Fig. 6a).^59^ The resulting [3]CPP^3^A is unstable and transforms into unknown insoluble species upon exposure to air. Interestingly, the stability of [3]CPP^3^A can be increased upon binding with C_70_. The complex, [3]CPP^3^A⊃C_70_ is stable in open air for more than three months without decomposition. With the stabilizing effect, the crystal structure of [3]CPP^3^A⊃C_70_ was obtained, which supports the CPPA molecular structure. On the contrary, C_60_ did not show strong binding with [3]CPP^3^A, neither the stabilizing effect. Alkyne bond angles (∠C–C≡C = 164°, 165°, and 168°) in [3]CPP^3^A molecular belts deviate from the normal 180° angle. Therefore, [3]CPP^3^A readily undergoes strain-promoted azide–alkyne cycloaddition (SPAAC), so-called copper-free click reaction, to form tris-triazoloparaterphenylene macrocycle **21**. Later in 2019, Lee and co-workers reported a follow-up work on the CPPA molecule synthesis.^60^ By utilizing the similar strategy, they successfully synthesized [3]CPP^4^A and [3]CPP^5^A (Fig. 6b) *via* alkyne metathesis followed by reductive aromatization. The synthesis of CPPA molecules enlightens a pathway of converting planar architectures to molecular belts, which would have great potential in the synthesis of artificial carbon nanobelts or nanotubes. However, the stabilization of CPPA molecules, enhancing the solubility, and the large-scale synthesis remains challenging.

## 4. Synthesis of shape-persistent ethynylene-linked organic molecular cages

Molecular cages have been the subject of intense research because of their unique topologies as well as properties. Ethynylene-linked organic cages, especially aryleneethynylene cages, have attracted great attention due to their shape-persistency and conjugated backbone structures, which often lead to interesting host–guest interactions and optoelectronic properties. As previously mentioned, thermodynamically controlled alkyne metathesis can provide ethynylene-linked shape-persistent macrocycles in one step in high yields. However, ethynylene-linked organic cages are still rare mainly due to the complexity of forming such closed three-dimensional (3D) structures and limited availability of suitable catalysts and monomer geometries. It should be noted that alkyne metathesis is not directional, and alkynes can self-react. Therefore, monomers of ethynylene-linked cages should be designed with preorganized bond angles and preferably symmetrical alkyne end groups to minimize heterogeneity of the products. In addition, unlike the synthesis of macrocycles, the productive pathway for the cage formation generally involves much higher strain energy barriers due to the confined 3D cage structures, requiring highly active and long lifetime alkyne metathesis catalysts. Thus, ethynylene-linked molecular cage synthesis has been one of the most challenging subjects in alkyne metathesis research area. Two main strategies have been explored for constructing molecular cages *via* alkyne metathesis: “Panel-directed”, forming the panel first, followed by cyclooligomerization, and “Vertex-directed”, forming the vertices first, followed by intramolecular metathesis. The topologies of molecular cages still remain largely unexplored due to the challenges in monomer synthesis, as well as unexpected reaction pathways. For the monomer design, if the panels are formed first, the panel sizes directly influence the energy landscape and kinetic factors along the reaction pathway, thus determining the final composition of the equilibrated system; if the vertices are synthesized first, the bite angle between each pedal will be an important parameter, which determines the feasibility of forming the target cages. Although alkyne metathesis faces many limitations in the synthesis of ethynylene-linked cages, it has rapidly emerged as a powerful synthetic tool to form purely organic cage products in high yields in one step.

**Fig. 7:**
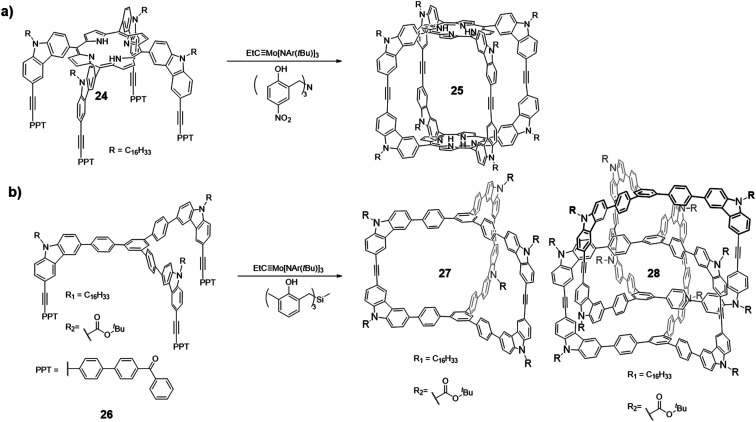
(a) Synthesis of COP-5 (cage **25**). (b) Synthesis of dimer/interlocked cages **27**/**28**. Reproduced with permission: (a) from ref. 27, Copyright 2011, The American Chemical Society; (b) from ref. 21, Copyright 2015, Wiley.

In 2011, Zhang and co-workers successfully synthesized the first ethynylene-linked organic cage (Covalent Organic Polyhedron, COP) through alkyne metathesis, namely COP-5 (Fig. 7a, cage **25**).^27^ The COP-5 has a rectangular prism topology containing two porphyrin rings as the top and bottom panels and carbazoles as the pillar part. The near 90° geometry of 3,6-substituted carbazole in the monomer **24** minimizes the angle strain in the resulting cage. Due to the multiple reaction sites and rigidity of the intermediates and the desired structure, a highly active alkyne metathesis catalyst with a long lifetime is crucial for this cage synthesis. Precipitation-driven alkyne metathesis of the monomer **24** was performed at 75 °C in CCl_4_ using trisbenzylamine-based multidentate Mo(vi) carbyne catalyst (**V-a**, R_1_ = H, R_2_ = NO_2_) to form COP-5. Meanwhile, the monodentate analogue of the catalyst (**III**) failed to provide the desired cage, only forming oligomeric species with a broad molecular weight distribution. The resulting COP-5 could be purified *via* column chromatography. MALDI-TOF shows the desired dimer *m*/*z* peak, and the effective hydrodynamic radius of COP-5 obtained from DOSY experiment is in good agreement with the simulated energy-minimized structure, which both support the formation of the desired cage.

In 2015, Zhang and co-workers reported the formation of a permanently interlocked cage through alkyne metathesis (Fig. 7b).^21^ Unlike the previous examples of imine- or boronate-linked interlocked cage formation, in which the products precipitated out of the reaction solution (kinetically trapped),^61–64^ the ethynylene-linked interlocked cage was formed in a homogeneous solution under thermodynamic control. Two tripodal monomers **26** with *C*_3_ symmetry, which have alkyl chains and Boc groups, respectively, as the substituents on the carbazole were designed. The alkyl chains were attached to improve the intermediates' solubility, while the Boc groups were designed to facilitate the separation of the pure dimer cage **27** and interlocked cage **28** through the polarity difference. The monomer with the electron-withdrawing Boc groups showed low reactivity and only formed oligomers when trisbenzylamine-based catalyst (**V-a**, R_1_ = H, R_2_ = NO_2_) was used. The desired mixture of dimer/interlocked cage **27**/**28** could only be obtained when trisbenzylsilane-based catalyst (**V-c**, R_1_ = Me, R_2_ = H) with a higher reactivity was used. This example again illustrates the activity of alkyne metathesis catalyst is crucial in the formation of cage structures, particularly for cages with electron-withdrawing groups. Due to the similar polarities between dimer/interlocked cages prepared from the alkyl-substituted monomers, they cannot be well separated, thus the ratio of dimer/interlocked cages cannot be determined. On the other hand, the dimer/interlocked cages substituted with the Boc groups with a higher polarity can be isolated *via* column chromatography. The ratio of dimer/interlocked (∼1 : 10 weight ratio) cages did not change after 24 h, which indicates this process reached an equilibrium and was under thermodynamic control. The ratio of dimer/interlocked cages were also influenced by the initial concentration of the monomer **26**, in which a higher concentration gave a higher ratio of the interlocked cage **28**. Interestingly, when using toluene as the solvent for alkyne metathesis, a significantly higher ratio of dimer cage **27** was observed. In addition, when the interlocked cage **28** was resubjected to the alkyne metathesis condition in toluene, it was partially converted to the dimer cage **27**, suggesting the possible templating effect of toluene solvent. This work demonstrates the first example of an interlocked cage structure assembled solely in solution phase. The studies on catalyst activity, monomer concentration, and solvent effect in alkyne metathesis also provide useful guidance for the future research.

**Fig. 8:**
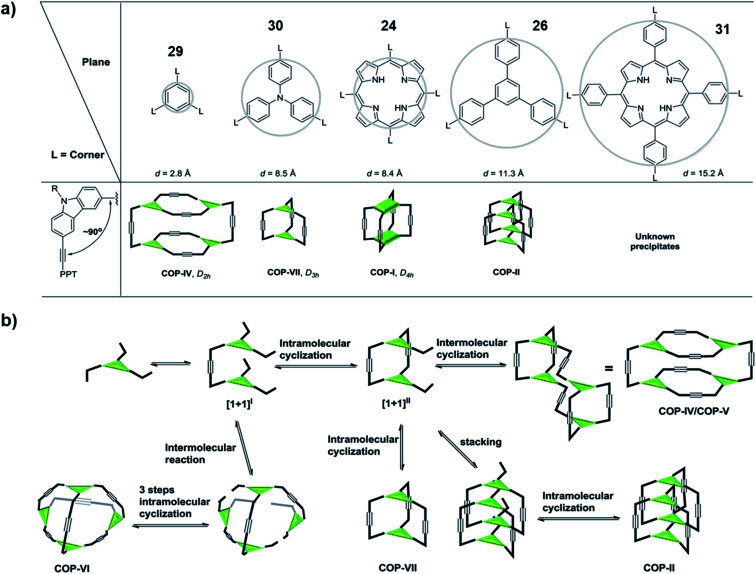
(a) The relationship between panel sizes and resulting product. (b) The proposed reaction pathway in cage formation. Reproduced with permission from ref. 65, Copyright 2016, The Royal Society of Chemistry.

In order to come up with a general guideline for the synthesis of ethynylene-linked cage formation, Zhang and co-workers conducted a systematic study using different building blocks (Fig. 8a).^65^ Based on their previous work on the synthesis of cubic cage **25**, *D*_2h_ cage **8**, and interlocked cage **28**, they further designed a tripodal phenyl- or triphenylamine-based monomers (**29** and **30**) and a tetrapodal expanded-porphyrin-based monomer **31** for comparison study. For the triphenylamine-based monomer **30**, they could successfully obtain the trigonal prismatic cage (COP-VII) under alkyne metathesis condition without tetramers or interlocked cages formed. For the expanded-porphyrin-based monomer **31**, however, due to the extremely low solubility of the resulting solids, no conclusion could be drawn from this monomer. They proposed the reaction pathway for the cage formation, and possible rationale for each product formation case by case (Fig. 8b). Basically, all the monomers first form dimers [1 + 1]^I^ through alkyne metathesis. Then there could be either intramolecular (formation of [1 + 1]^II^) or intermolecular alkyne metathesis reaction pathway. If there is no large steric hinderance, all the monomers tend to go through the intramolecular reaction to form [1 + 1]^II^, which could explain the failed synthesis of the *T*_d_ cage (COP-VI). If the productive pathway for the last alkyne metathesis step (cage formation by closing the last side arm) does not experience a high energy barrier, the monomer would form a dimeric cage (COP-VII) as the desired product. However, at this stage, if the panel of the cage is large, there is a higher possibility that two intermediates stacked together to form an interlocked structure (COP-II), *e.g.*, **28** in Fig. 7b. On the other hand, if the panel is too small (*e.g.*, the product from the monomer **29** or **7**), the productive pathway would be kinetically disfavored (causing significant angle strain). This would cause the dimeric intermediate to further dimerize, leading to the formation of a tetrameric cage (COP-IV/COP-V) *e.g.*, **8** in Fig. 2. This work proposed a plausible reaction pathway for the formation of dimer, tetramer, interlocked or polymer products consisting of different panels, which highlights the important role of the panel size in cage formation.

**Fig. 9:**
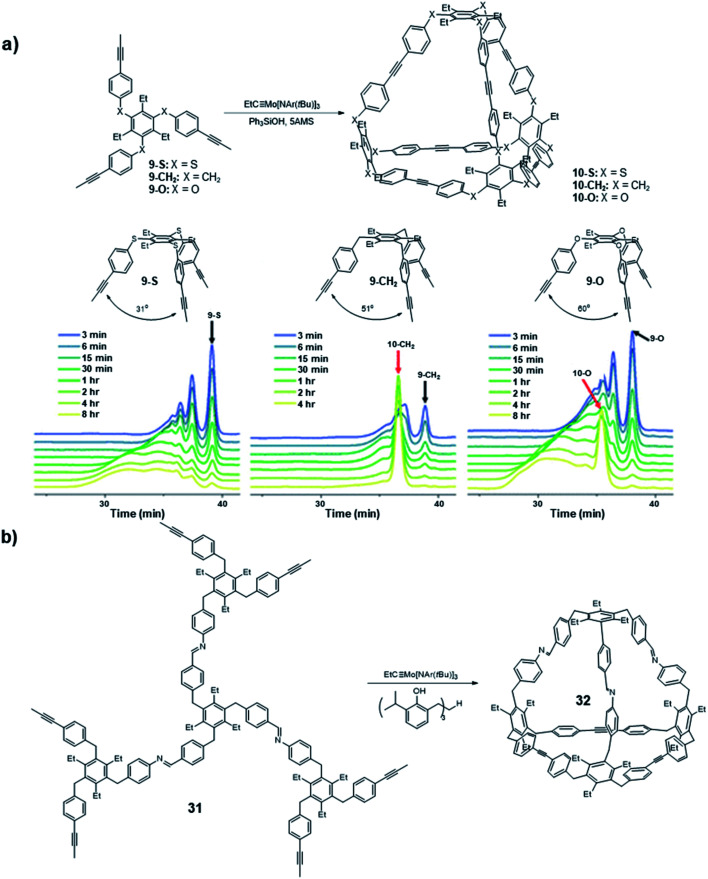
(a) The GPC traces of different monomers with different bite angles. (b) The *C*_3v_ cage **32** synthesized by Moore and co-workers with a responsive vertex. Reproduced with permission: (a) from ref. 66, Copyright 2018, The American Chemical Society; (b) from ref. 22, Copyright 2019, The Royal Society of Chemistry.

Following the synthesis of the *T*_d_ symmetric cage **10** that is kinetically trapped, a systematic study focused on the bite angle of the precursors was conducted by Moore and co-workers (Fig. 9a).^66^ Specifically, they studied the influence of the bite angle on the cage formation. They used similar tripodal precursors (**9-S**, **9-CH2**, **9-O**) in this study, but changed the bite angle to 31°, 51°, and 60° by varying the connecting atoms (sulfur, carbon, and oxygen, respectively) between the benzene rings. The three monomers were subjected to the alkyne metathesis individually with the catalyst generated *in situ* by mixing the Mo precursor (**II**) with Ph_3_SiOH. The carbon linked monomer **9-CH2** showed the same nearly quantitative conversion to the cage **10-CH2** as in the previous study; the oxygen linked monomer **9-O** showed the formation of tetrahedral cage **10-O** along with some oligomers after 8 h based on GPC; the sulfur linked monomer **9-S** did not show the explicit signal corresponding to the tetrahedral cage, and only oligomers were observed. Although the monomer **9-O** has the closest bite angle to the ideal tetrahedral structure, the convergence rate to the cage **10-O** was much slower than that of **9-CH2**. Nevertheless, it eventually formed the cage near quantitative yield after 24 h. This study shows monomers with a slightly tight bite angle can form the tetrahedral cage more efficiently. They also used the combinations of those monomers in equal molar ratio in alkyne metathesis to incorporate the monomer **9-S** in the cage structure. All the possible structures except cage **10-S** composed solely of **9-S** monomer were observed, suggesting that the monomers with loose bite angles release the angle strain and enable the scrambling but do not prompt self-sorting. This study demonstrates that subtle differences in the monomer geometry can significantly alter the energy landscape of the reaction pathway, ultimately influencing the outcome of the dynamic assembly process.

A similar cage but with responsive imine vertices was synthesized through the orthogonal strategy by Moore and co-workers in 2019 (Fig. 9b).^22^ They designed a tripodal monomer **31** with imine linkages to form the cage **32** with *C*_3v_ symmetry. The hexatopic monomer **31** was prepared *via* trifold imine condensation, which exhibits decent solubility in conventional alkyne metathesis reaction solvents (CHCl_3_, CCl_4_) and can be easily purified *via* precipitation in methanol. Due to the imine moieties in the molecule, a highly active Mo(vi)-based multidentate catalyst (**V-d**, R_1_ = ^i^Pr, R_2_ = H) was used instead of the monodentate ligand-based catalyst generated *in situ* by mixing the Mo precursor (**II**) with Ph_3_SiOH. The resulting cage **32** has a responsive vertex, which could be activated under imine exchange condition. This study, for the first time, synthesized a cage linked by imine and ethynylene linkages. Moreover, it demonstrates the strategy for preparing cages with altered symmetry, opening new possibilities for synthesizing molecular cages with greater control over symmetry and functionality.

## 5. Applications of aryleneethynylene-based molecular architectures

**Fig. 10:**
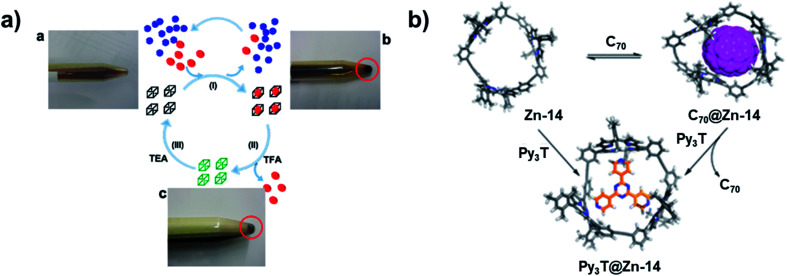
(a) Selective reversible fullerene-binding of cage **25** for fullerene purification. (b) Porphyrin-based trimers **14** with host–guest exchange nature. Reproduced with permission: (a) from ref. 27, Copyright 2011, The American Chemical Society; (b) from ref. 56, Copyright 2016, The American Chemical Society.

Besides the novel structures themselves, the intriguing properties of aryleneethynylene molecular architectures have also attracted great interest. The ethynylene linkages enable large conjugation in those architectures, which provides unique properties in host–guest binding, light-harvesting, chemical sensing, and ion-transportation. Given the electron-rich nature and confined internal cavity, shape-persistent aryleneethynylene cages can serve as excellent host molecules for electron acceptors, *e.g.*, fullerenes, with a high binding selectivity. For example, ethynylene-linked shape-persistent cage **25** reported by Zhang and co-workers in 2011 shows selective binding toward different fullerenes.^27^ The cage cavity can strongly bind C_70_ and C_60_ by forming a 1 : 1 complex. Moreover, due to the difference in the guest size and structure rigidity of the cage host, the cage molecule shows highly selective (1000 times difference) binding of C_70_ (*K*_assoc_ = 1.5 × 10^8^ M^−1^) over C_60_ (*K*_assoc_ = 1.4 × 10^5^ M^−1^) in toluene. It is noteworthy that this is the first demonstration of using non-metallated porphyrin moieties for selective fullerene binding. The non-metallated nature of cage **25** also enables the reversible association/dissociation process of fullerenes by protonating/deprotonating the porphyrin moieties. Upon treatment of the fullerene–cage complex with excess amount of trifluoroacetic acid (TFA, 100 eq.), the complete release of fullerene molecules was observed; while upon further neutralization with triethylamine (TEA) of the mixture above, the cage-fullerene binding interactions were restored. The reversible binding of fullerenes enables practical fullerene purification: cage **25** can selectively extract C_70_ from the C_60_-enriched C_60_/C_70_ mixture in CS_2_. The excess free fullerenes can be precipitated out in CHCl_3_, and the solution phase can be acidified to release C_70_ bound inside the cage **25**. The C_70_ abundance was increased from 9% to 79% after only one purification cycle (Fig. 10a). This work provides a proof-of-concept for the practical purification of fullerene mixtures using by-design cage molecules. Later, Zhang and co-workers also synthesized a porphyrin-based macrocycle **12** with similar size as cage **25**.^56^ Due to the open side of the macrocycle, the macrocycle **12** exhibited a stronger binding interaction with the larger fullerene molecule C_84_. This macrocycle showed over 1500 times stronger binding interaction with C_84_ (*K*_assoc_ = 2.2 × 10^7^ M^−1^) over C_60_ (*K*_assoc_ = 1.3 × 10^4^ M^−1^), which also has the reversible association/dissociation binding feature and could be further applied to fullerene purification applications. Similarly, a porphyrin-based trimeric macrocycle **14** can also bind fullerenes,^57^ however, showing only moderate binding affinity for C_70_ (*K*_assoc_ = 6 × 10^3^ M^−1^). It does not have explicit binding interaction with C_60_ or C_84_, thus still exhibiting a relatively high binding selectivity for C_70_ over other fullerene molecules (Fig. 10b). In addition, the metalated macrocycle, Zn-**14**, showed a strong binding affinity with 2,4,6-tri(4-pyridyl)-1,3,5-triazine (Py_3_T, *K*_assoc_ = 4 × 10^9^ M^−1^). Upon the addition of Py_3_T to C_70_@**14**, C_70_ was fully released, indicating the reversible binding of C_70_ with macrocycle **14**. Other than the porphyrin-based macrocycles, aromatic-rich macrocycles or cages also show selective binding with fullerene molecules. The *D*_2h_ symmetric cage **8** showed a moderate binding with C_70_ (*K*_assoc_ = 3.9 × 10^3^ M^−1^), but no binding interactions with C_60_.^44^

**Fig. 11:**
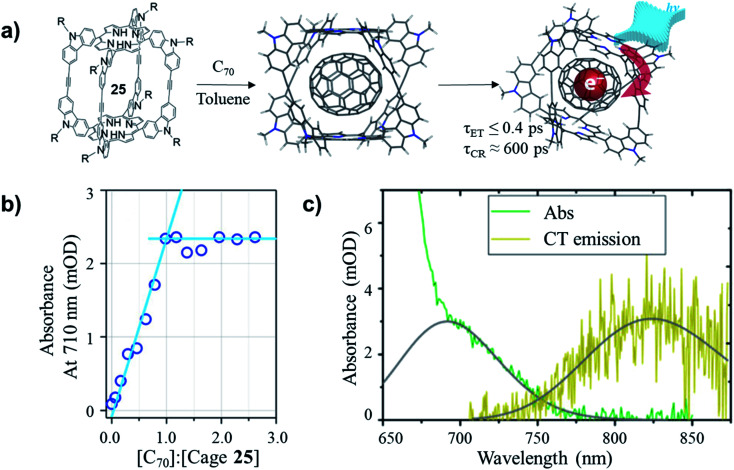
(a) Formation and photoinduced electron transfer for C_70_@**25** complex. (b) Absorbance at 710 nm as a function of C_70_/**25** molar ratio. (c) Comparative spectra (green and yellow) with (gray) Gaussian fits of (green) absorption for C_70_/**25** (molar ratio of 2.0) and (yellow) CT emission for C_70_/**25** (≈10 molar ratio), in steady-state photoluminescence experiments, ∼10 : 1 molar ratio of C_70_/**25** is used to suppress unbound cage **25** emission. Reproduced with permission from ref. 67, Copyright 2017, The American Chemical Society.

The non-covalently bound C_70_–cage **25** hybrid structure was later found possessing ultrafast photoinduced electron transfer (PIET) dynamics in a low-dielectric medium.^67^ The stoichiometry of C_70_ and cage **25** in the complex was confirmed as 1 : 1 through the titration in toluene. As shown in Fig. 11a and b, before the 1 : 1 ratio of [C_70_]/[cage **25**] was reached, the absorbance at 710 nm increased linearly with [C_70_]/[cage **25**] ratio, while the absorbance remained constant after the ratio reached 1.0. Based on the comparative spectra of the absorption and charge transfer (CT) emission (Fig. 11c), the Gibbs energy of charge recombination was determined to be 
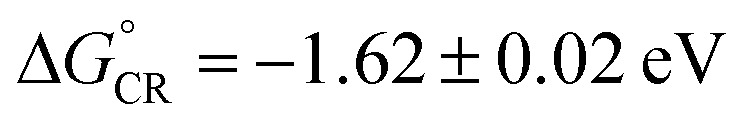
 and internal reorganization energy to be *λ*_i_ = 0.13 ± 0.03 eV. Ultrafast PIET time constant (*τ*_ET_ ≤ 0.4 ps) and very slow charge recombination (*τ*_CR_ ≈ 600 ps) could be achieved using structurally rigid donor–acceptor molecular hybrid C_70_@**25** complex in toluene. This work reveals that the donor–acceptor coupling in a rigid structural entity could be a promising strategy to realize high *τ*_CR_/*τ*_ET_ ratio.

**Fig. 12:**
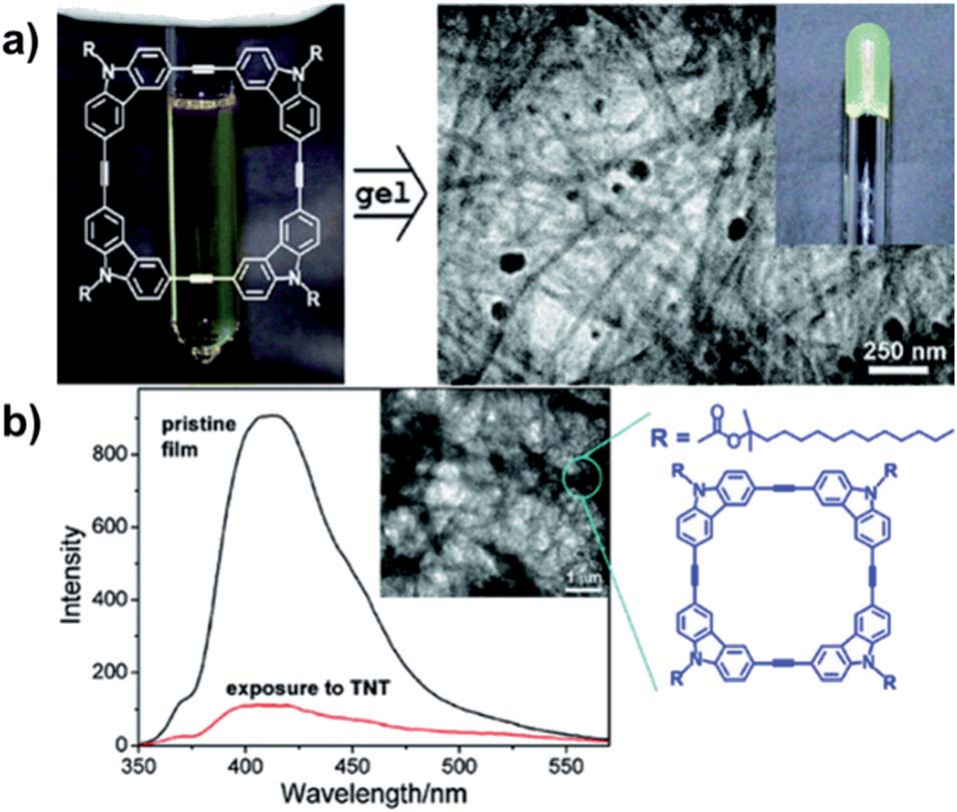
(a) Fabrication of macrocycle-based nanofibril upon gelation. (b) The fluorescence quenching of the nanofibril film after exposure to TNT. Reproduced with permission: (a) from ref. 68, Copyright 2006, The American Chemical Society; (b) from ref. 69, Copyright 2007, The American Chemical Society.

The electron-rich nature of aryleneethynylene structures enables the ready formation of D–A complexes with charge-transfer-induced fluorescence quenching effect, which can have potential sensing applications. In 2006, Zang and Moore reported the nanofibril self-assembly of the aryleneethynylene macrocycles by spin-casting a cyclohexane solution of the macrocycle (Fig. 12a).^68^ The obtained nanofibril film exhibited strong fluorescence with a quantum yield of 0.19.^69^ The film showed decent efficiency of fluorescence quenching after 60 s exposure in the vapor of 2,4-dinitrotoluene (DNT) (90%) or 2,4,6-trinitrotoluene (TNT) (83%) (Fig. 12b). The quenching efficiency of this nanofibril film was nearly independent on the thickness of the film due to its porous nature, which is in great contrast to the previous works, where the thicker film usually gave much lower quenching efficiency.^70,71^ In addition, the fluorescence could be recovered to 90% after exposing the film in the saturated vapor of hydrazine for 1 h. The fluorescence quenching-recovery cycle did not change too much within five cycles. By fabricating the aryleneethynylene macrocycles into a nanofibril film on a surface, a new type of fluorescence sensory material could be developed, which could potentially be utilized in explosive sensing.

**Fig. 13:**
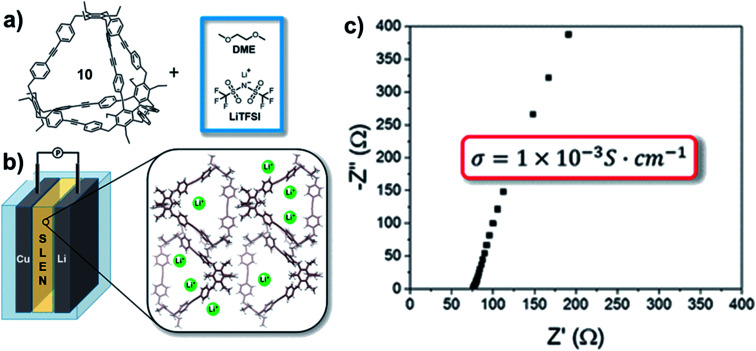
(a) Fabrication of SLENs. (b) The structure of the half-cell with SLENs. (c) The conductivity of the resulting SLENs at room temperature. Reproduced with permission from ref. 72, Copyright 2018, The American Chemical Society.

Aryleneethynylene cages, due to their shape-persistency and porosity, can also serve as the ion-transportation medium in solid-state lithium battery. The tetrahedral cage **10-CH2** synthesized by Moore and co-workers^45^ was mixed with a 1 M bis(trifluoromethane)-sulfonamide lithium salt (LiTFSI) solution in 1,2-dimethoxyethane (DME) to form a solid–liquid electrolyte nanocomposite (SLENs) (Fig. 13a).^72^ The obtained SLENs was tested for their ion-transportation property (Fig. 13b). At room temperature, the SLENs showed a high Li^+^ conductivity of 1.0 ± 0.1 × 10^−3^ S cm^−1^ (Fig. 13c), which is higher than many MOFs, COFs, and porous organic based SLENs. The Arrhenius plot shows the activation energy as low as 16 kJ mol^−1^ at the temperature ranging from −10 °C to 45 °C. However, the significant decomposition of this SLENs at temperature higher than 45 °C impedes its practical applications. Full-cell linear sweep voltammogram measurement showed the SLENs are stable up to 4.7 V. To further demonstrate the key role of the shape-persistent cage, the monomer of this cage was also tested under the same condition. The monomer showed much higher resistance and much lower transference number, which indicates the advantages of the porous organic cages. This work opens the possibility of utilizing porous shape-persistent cages in battery-related research.

The applications of the ethynylene-linked molecular architectures have been mainly based on their host–guest interaction, large conjugation system, and shape-persistency. Compared with polymers, the discrete nature of these architectures makes them easier to get crystalline or even single-crystal structures, which greatly assists their structure determination and the host–guest interaction study at the atomic level. Moreover, the decent solubility of these molecular architectures, compared to insoluble polymers, provides better interactions with guest molecules or probe species. The large conjugation, high aromaticity, and carbon-rich nature could enable their potential applications in various fields, such as molecular separation, optoelectronic devices, chemical sensing, *etc.*

## 6. Conclusions and outlook

Within the past few decades, there have been copious efforts dedicated to the development of aryleneethynylene macrocycles and cages, including the development of catalysts, optimizing end groups, novel backbones, and innovative applications: several highly active multidentate catalysts have been developed; 1-pentynyl group shows great potential in serving as an optimal end group due to its decent solubility and easy handling; a series of novel macrocycles and cages have been synthesized, including Möbius ring structure, CPPA structure, *C*_3v_, *D*_2h_, *D*_3h_, *D*_4h_, *T*_d_, and interlocked structures; applications in selective fullerene binding, light harvesting, explosive sensing, and solid electrolyte were also demonstrated. Compared with conventional irreversible cross-coupling reactions, alkyne metathesis can provide desired products in one step in high yields, which can significantly shorten the synthetic routes and enhance the reaction economy. However, monomers for alkyne metathesis have more stringent requirements than cross-coupling substrates: for example, the monomers generally cannot have strong acidic group (*e.g.*, –COOH) or strong chelating groups (*e.g.*, α-pyridyl, –NH_2_); the panel size or the bite angle should be judiciously designed; the solubility of the possible intermediates should be maintained to avoid precipitation; the highly active catalyst is required for certain “tough” (less reactive) substrates. Although ethynylene-linked macrocycles and cages could be obtained by cross-coupling reactions in low yields, alkyne metathesis is highly preferred due to its unique self-correction character.

Although significant progress has been achieved, several challenges remain. Highly active and long lifetime catalysts are still on high demand for synthesis of challenging structures. Specifically, the catalysts compatible with chelating, protic, or strong electron-withdrawing groups are still rare. The energy landscape and kinetic factors of the dynamic system are highly sensitive to monomer structures and geometry, making the monomer design and prediction of desired structures difficult. DFT calculations on the possible intermediates could be helpful to further fine tuning of monomer structures to promote the formation of specific target structures. Kinetic traps can be used as more strategic ways to obtain complicated structures that are not thermodynamically favored. On the other hand, in scenarios involving kinetic traps, we can introduce driving forces (*e.g.*, template effect by chelating or donor–acceptor interactions) to obtain target products. Alkyne metathesis is a homo-coupling reaction, which does not have the directionality like imine condensation. Therefore, the symmetrical installation of the end groups is preferred to avoid undesired products, which limits the possibilities of synthesizing structures with lower symmetries. Applications of these intriguing structures have still been limited, and many other applications stemming from their conjugated structures, shape-persistency, well-defined internal cavities are anticipated.

## Author contributions

S. H. and W. Z. prepared the review outline. All the authors participated in writing and revising the manuscript.

## Conflicts of interest

There are no conflicts to declare.

## References

[cit1] Jin Y., Yu C., Denman R. J., Zhang W. (2013). Chem. Soc. Rev..

[cit2] Zhang W. and Jin Y., Dynamic Covalent Chemistry: Principles, Reactions, and Applications, Wiley-VCH, Weinheim, 2017

[cit3] Pennella F., Banks R. L., Bailey G. C. (1968). Chem. Commun..

[cit4] Fürstner A. (2013). Angew. Chem., Int. Ed..

[cit5] Ehrhorn H., Tamm M. (2019). Chem.–Eur. J..

[cit6] Zhang W., Moore J. S. (2007). Adv. Synth. Catal..

[cit7] Benson S., Collin M.-P., Arlt A., Gabor B., Goddard R., Fürstner A. (2011). Angew. Chem., Int. Ed..

[cit8] Valot G., Regens C. S., O'Malley D. P., Godineau E., Takikawa H., Fürstner A. (2013). Angew. Chem., Int. Ed..

[cit9] Roland C. D., Li H., Abboud K. A., Wagener K. B., Veige A. S. (2016). Nat. Chem..

[cit10] Fischer F. R., Nuckolls C. (2010). Angew. Chem., Int. Ed..

[cit11] Paley D. W., Sedbrook D. F., Decatur J., Fischer F. R., Steigerwald M. L., Nuckolls C. (2013). Angew. Chem., Int. Ed..

[cit12] Bellone D. E., Bours J., Menke E. H., Fischer F. R. (2015). J. Am. Chem. Soc..

[cit13] von Kugelgen S., Piskun I., Griffin J. H., Eckdahl C. T., Jarenwattananon N. N., Fischer F. R. (2019). J. Am. Chem. Soc..

[cit14] Yang H., Jin Y., Du Y., Zhang W. (2014). J. Mater. Chem. A.

[cit15] Ge P.-H., Fu W., Herrmann W. A., Herdtweck E., Campana C., Adams R. D., Bunz U. H. F. (2000). Angew. Chem., Int. Ed..

[cit16] Miljanić O. Š., Vollhardt K. P. C., Whitener G. D. (2003). Synlett.

[cit17] Zhang W., Kraft S., Moore J. S. (2003). Chem. Commun..

[cit18] Zhang W., Kraft S., Moore J. S. (2004). J. Am. Chem. Soc..

[cit19] Heppekausen J., Stade R., Goddard R., Fürstner A. (2010). J. Am. Chem. Soc..

[cit20] Bindl M., Stade R., Heilmann E. K., Picot A., Goddard R., Fürstner A. (2009). J. Am. Chem. Soc..

[cit21] Wang Q., Yu C., Long H., Du Y., Jin Y., Zhang W. (2015). Angew. Chem., Int. Ed..

[cit22] Pattillo C. C., Moore J. S. (2019). Chem. Sci..

[cit23] Jyothish K., Zhang W. (2011). Angew. Chem., Int. Ed..

[cit24] Jyothish K., Zhang W. (2011). Angew. Chem., Int. Ed..

[cit25] Yang H., Liu Z., Zhang W. (2013). Adv. Synth. Catal..

[cit26] Du Y., Yang H., Zhu C., Ortiz M., Okochi K. D., Shoemaker R., Jin Y., Zhang W. (2016). Chem.–Eur. J..

[cit27] Zhang C., Wang Q., Long H., Zhang W. (2011). J. Am. Chem. Soc..

[cit28] Beer S., Hrib C. G., Jones P. G., Brandhorst K., Grunenberg J., Tamm M. (2007). Angew. Chem., Int. Ed..

[cit29] Beer S., Brandhorst K., Hrib C. G., Wu X., Haberlag B., Grunenberg J., Jones P. G., Tamm M. (2009). Organometallics.

[cit30] Estes D. P., Bittner C., Àrias Ò., Casey M., Fedorov A., Tamm M., Copéret C. (2016). Angew. Chem., Int. Ed..

[cit31] Estes D. P., Gordon C. P., Fedorov A., Liao W.-C., Ehrhorn H., Bittner C., Zier M. L., Bockfeld D., Chan K. W., Eisenstein O., Raynaud C., Tamm M., Copéret C. (2017). J. Am. Chem. Soc..

[cit32] Hillenbrand J., Leutzsch M., Fürstner A. (2019). Angew. Chem., Int. Ed..

[cit33] Thompson R. R., Rotella M. E., Du P., Zhou X., Fronczek F. R., Kumar R., Gutierrez O., Lee S. (2019). Organometallics.

[cit34] Hillenbrand J., Leutzsch M., Yiannakas E., Gordon C. P., Wille C., Nöthling N., Copéret C., Fürstner A. (2020). J. Am. Chem. Soc..

[cit35] Cui M., Bai W., Sung H. H. Y., Williams I. D., Jia G. (2020). J. Am. Chem. Soc..

[cit36] Ge Y., Huang S., Hu Y., Zhang L., He L., Krajewski S., Ortiz M., Jin Y., Zhang W. (2021). Nat. Commun..

[cit37] Rowan S. J., Cantrill S. J., Cousins G. R. L., Sanders J. K. M., Stoddart J. F. (2002). Angew. Chem., Int. Ed..

[cit38] Yang H., Du Y., Wan S., Trahan G. D., Jin Y., Zhang W. (2015). Chem. Sci..

[cit39] Kiel G. R., Bergman H. M., Tilley T. D. (2020). Chem. Sci..

[cit40] Kiel G. R., Bay K. L., Samkian A. E., Schuster N. J., Lin J. B., Handford R. C., Nuckolls C., Houk K. N., Tilley T. D. (2020). J. Am. Chem. Soc..

[cit41] Zhang W., Moore J. S. (2004). J. Am. Chem. Soc..

[cit42] Zhang W., Brombosz S. M., Mendoza J. L., Moore J. S. (2005). J. Org. Chem..

[cit43] Zhang W., Moore J. S. (2005). J. Am. Chem. Soc..

[cit44] Wang Q., Zhang C., Noll B. C., Long H., Jin Y., Zhang W. (2014). Angew. Chem., Int. Ed..

[cit45] Lee S., Yang A., Moneypenny T. P., Moore J. S. (2016). J. Am. Chem. Soc..

[cit46] Elliott E. L., Hartley C. S., Moore J. S. (2011). Chem. Commun..

[cit47] Ponnuswamy N., Cougnon F. B. L., Clough J. M., Pantoş G. D., Sanders J. K. M. (2012). Science.

[cit48] Kim J.-K., Lee E., Kim M.-C., Sim E., Lee M. (2009). J. Am. Chem. Soc..

[cit49] Rondeau-Gagné S., Néabo J. R., Desroches M., Larouche J., Brisson J., Morin J.-F. (2013). J. Am. Chem. Soc..

[cit50] Wang Q., Zhong Y., Miller D. P., Lu X., Tang Q., Lu Z.-L., Zurek E., Liu R., Gong B. (2020). J. Am. Chem. Soc..

[cit51] Fischer M., Lieser G., Rapp A., Schnell I., Mamdouh W., De Feyter S., De Schryver F. C., Höger S. (2004). J. Am. Chem. Soc..

[cit52] Höger S. (2010). Pure Appl. Chem..

[cit53] Diederich F., Kivala M. (2010). Adv. Mater..

[cit54] Zang L., Che Y., Moore J. S. (2008). Acc. Chem. Res..

[cit55] Zhang W., Moore J. S. (2006). Angew. Chem., Int. Ed..

[cit56] Zhang C., Long H., Zhang W. (2012). Chem. Commun..

[cit57] Yu C., Long H., Jin Y., Zhang W. (2016). Org. Lett..

[cit58] Jiang X., Laffoon J. D., Chen D., Pérez-Estrada S., Danis A. S., Rodríguez-López J., Garcia-Garibay M. A., Zhu J., Moore J. S. (2020). J. Am. Chem. Soc..

[cit59] Lee S., Chénard E., Gray D. L., Moore J. S. (2016). J. Am. Chem. Soc..

[cit60] Zhou X., Thompson R. R., Fronczek F. R., Lee S. (2019). Org. Lett..

[cit61] Hasell T., Wu X., Jones J. T. A., Bacsa J., Steiner A., Mitra T., Trewin A., Adams D. J., Cooper A. I. (2010). Nat. Chem..

[cit62] Zhang G., Presly O., White F., Oppel I. M., Mastalerz M. (2014). Angew. Chem., Int. Ed..

[cit63] Beves J. E., Leigh D. A. (2010). Nat. Chem..

[cit64] Ji Q., Lirag R. C., Miljanić O. Š. (2014). Chem. Soc. Rev..

[cit65] Wang Q., Yu C., Zhang C., Long H., Azarnoush S., Jin Y., Zhang W. (2016). Chem. Sci..

[cit66] Moneypenny T. P., Yang A., Walter N. P., Woods T. J., Gray D. L., Zhang Y., Moore J. S. (2018). J. Am. Chem. Soc..

[cit67] Ortiz M., Cho S., Niklas J., Kim S., Poluektov O. G., Zhang W., Rumbles G., Park J. (2017). J. Am. Chem. Soc..

[cit68] Balakrishnan K., Datar A., Zhang W., Yang X., Naddo T., Huang J., Zuo J., Yen M., Moore J. S., Zang L. (2006). J. Am. Chem. Soc..

[cit69] Naddo T., Che Y., Zhang W., Balakrishnan K., Yang X., Yen M., Zhao J., Moore J. S., Zang L. (2007). J. Am. Chem. Soc..

[cit70] McQuade D. T., Pullen A. E., Swager T. M. (2000). Chem. Rev..

[cit71] Toal S. J., Trogler W. C. (2006). J. Mater. Chem..

[cit72] Petronico A., Moneypenny T. P., Nicolau B. G., Moore J. S., Nuzzo R. G., Gewirth A. A. (2018). J. Am. Chem. Soc..

